# *In situ* micro-focused X-ray beam characterization with a lensless camera using a hybrid pixel detector

**DOI:** 10.1107/S1600577513034759

**Published:** 2014-02-04

**Authors:** Anton Kachatkou, Julien Marchal, Roelof van Silfhout

**Affiliations:** a School of Electrical and Electronic Engineering, The University of Manchester, Sackville Street Building, Manchester M13 9PL, UK; b Detector Group, Diamond Light Source Ltd, Diamond House, Harwell Science and Innovation Campus, Didcot, Oxfordshire OX11 0DE, UK

**Keywords:** X-ray imaging, pinhole camera, beam diagnostics, micro-focus, scattering measurements, beam size measurements

## Abstract

Results of studies on micro-focused X-ray beam diagnostics using an X-ray beam imaging (XBI) instrument based on the idea of recording radiation scattered from a thin foil of a low-*Z* material with a lensless camera are reported. The XBI instrument captures magnified images of the scattering region within the foil as illuminated by the incident beam. These images contain information about beam size, beam position and beam intensity that is extracted during dedicated signal processing steps. In this work the use of the device with beams for which the beam size is significantly smaller than that of a single detector pixel is explored. The performance of the XBI device equipped with a state-of-the-art hybrid pixel X-ray imaging sensor is analysed. Compared with traditional methods such as slit edge or wire scanners, the XBI micro-focused beam characterization is significantly faster and does not interfere with on-going experiments. The challenges associated with measuring micrometre-sized beams are described and ways of optimizing the resolution of beam position and size measurements of the XBI instrument are discussed.

## Abstract 2

Position and size measurements of a micro-focused X-ray beam, using an X-ray beam imaging device based on a lensless camera that collects radiation scattered from a thin foil placed in the path of the beam at an oblique angle, are reported.

## 1. Introduction   

Tightly focused X-ray beams have been playing an increasingly important role in applications of synchrotron radiation. Beam sizes of a few micrometres are routinely obtained using Kirkpatrick–Baez mirrors (Kirkpatrick & Baez, 1948 ▶) and compound refractive lenses (Snigirev *et al.*, 1996 ▶) whereas diffraction-limited focusing with beam sizes in a deep sub-micrometre range can be achieved with Fresnel zone plates (Schroer, 2006 ▶). Such small beam sizes as well as the associated increase in beam intensity demand appropriate beam diagnostics tools to measure the beam’s size and position. Wire scanners (Fulton *et al.*, 1989 ▶) and beam monitors based on fluorescent screens (Fuchs *et al.*, 2007 ▶) are the most popular solutions. However, the former obstruct the beam and are relatively slow; the latter, while providing the desired versatility and ‘semi-transparency’, often suffer from slow deterioration of the fluorescent material and signal saturation due to the high intensity of the incident radiation.

In this paper, we report on *in situ* diagnostics of micro-focused X-ray beams performed with a recently proposed X-ray beam imaging (XBI) device (Kachatkou *et al.*, 2013 ▶; Kachatkou & van Silfhout, 2013 ▶; van Silfhout *et al.*, 2011 ▶). The instrument records X-ray radiation scattered from a thin foil of a low-*Z* material with a lensless (pinhole) camera. The low-*Z* material of the foil ensures that only a negligible amount of radiation is scattered so that the device is transparent to the incident beam and the signal recorded by the camera is typically significantly lower than the saturation threshold of the detector. In the subsequent sections we investigate the performance of the XBI instrument and analyse advantages and disadvantages of equipping the XBI instrument with a state-of-the-art hybrid pixel X-ray detector. Such detectors usually operate in a single-photon-counting mode and are capable of providing virtually noiseless images. However, their pixels are typically made relatively large in order to reduce X-ray-induced charge sharing between neighbouring pixels (Henrich *et al.*, 2009 ▶; Dinapoli *et al.*, 2011 ▶; Ballabriga *et al.*, 2011 ▶).

## 2. Micro-focused beam diagnostics   

X-ray beam diagnostics is typically concerned with measurements of beam position, intensity and size. Also, for a comprehensive analysis of the performance of the beamline’s upstream beam-shaping elements, it is beneficial to obtain images of the beam’s cross section (Kachatkou *et al.*, 2013 ▶). The difficulty associated with micro-focused beam diagnostics is the small beam size. For example, suitable scintillator screens have resolutions of no better than 20 µm, which makes it impossible for conventional beam monitors based on such screens to measure the size of beams only a few micrometres wide. We exploit the fact that the XBI device creates a magnified image of the beam so that such small beams can be imaged with a conventional pixelated area detector.

Beam position measurements with the XBI device are discussed in detail by Kachatkou & van Silfhout (2013 ▶). In brief, every image recorded by the instrument (XBI image) is converted into vertical and horizontal profiles by summing up image pixel values along each row and column, respectively. This procedure reduces the amount of data to be processed and significantly boosts the signal-to-noise ratio (SNR). Each profile is then fitted with a Gaussian function whose centre is the respective coordinate of the image centre. The difference between two consecutively measured coordinates is linearly related to the corresponding change in beam position (Kachatkou & van Silfhout, 2013 ▶).

**Figure 1:**
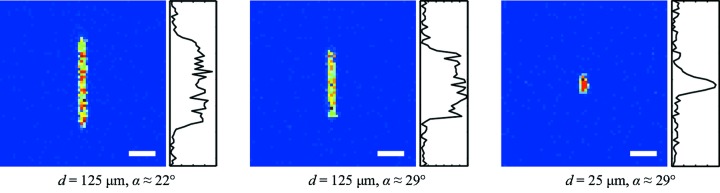
XBI images of the micro-focused X-ray beam (approximately 5 µm × 5 µm r.m.s.) taken using different scatter foil thicknesses *d* and tilt angles α; XBI magnification is 5; aperture is a 25 µm pinhole; scale bar is 550 µm.

The width of the Gaussian fitted to the XBI image profiles obtained as described above can also be used to detect changes in the beam’s size. However, the absolute measurements of the size of the incident beam are impeded by a number of factors that affect the shape and the size of the XBI images and, consequently, corresponding profiles. Firstly, XBI images are elongated along the direction of the foil tilt due to the combined effect of the foil thickness and the tilt angle (Fig. 1 ▶). Secondly, the device magnification varies along the direction of the foil tilt (Kachatkou *et al.*, 2013 ▶). Image blur caused by the shape and finite dimensions of the aperture and due to the detector point spread function (PSF) further increases the size and distorts the shape of XBI images. Hence, to measure the beam size, it is first necessary to remove the aforementioned distortions from a beam image, *i.e.* reconstruct a beam cross-sectional image. These distortions are modelled by the XBI PSF, and the associated image reconstruction algorithm is described by Kachatkou *et al.* (2013 ▶).

The light intensity distribution along the cross section of a focused X-ray beam can typically be approximated by a two-dimensional Gaussian function. Thus, the size of the beam can be characterized in the horizontal and vertical directions by two corresponding Gaussian widths. These two Gaussian widths are used as a measure of the beam size throughout this work. Consequently, the X-ray beam size is obtained from a reconstructed beam cross-sectional image by calculating its horizontal and vertical profiles and fitting those profiles to a Gaussian function.

## 3. Experiments   

The experiments were conducted at bending magnet test beamline B16 at the Diamond Light Source synchrotron facility (UK). A monochromatic 15 keV X-ray beam from the double-multilayer monochromator was shaped by 1 mm × 1 mm slits and focused by a beryllium parabolic compound refractive lens (CRL) built from 27 individual lenses with a radius of curvature of 0.2 mm, the thickness between the parabolas of the lens approximately 35 µm and the diameter of the lens aperture 1 mm. The distance between the source and the CRL was 43.6 m. The estimated flux through the input aperture of the CRL was 3 × 10^10^ photons s^−1^ and the calculated CRL transmission at 15 keV was approximately 42%. The CRL was creating an image of the source with a calculated size of 3.1 µm × 1.4 µm r.m.s. (h × v) (Sawhney *et al.*, 2010 ▶; Lengeler *et al.*, 1999 ▶).

The XBI device was equipped with an X-ray detector consisting of a single Medipix 3.0 readout chip (Ballabriga *et al.*, 2011 ▶) bump-bonded to a 300 µm-thick silicon photodiode sensing layer. Medipix 3.0 has 256 × 256 pixels, each with an area of 55 µm × 55 µm. The detector was used in a single-photon-counting mode with the charge-sharing correction logic disabled. For relatively small pixel sizes, such as with the Medipix 3.0 chip, charge-sharing between pixels introduces dependence of the imaging performance of the detector on its detection threshold. When using monochromatic X-rays, a threshold set at 50% of the energy gives optimum performance in terms of detective quantum efficiency (Marchal & Medjoubi, 2012 ▶). Therefore, in our experiments, the detection threshold was set at 7.5 keV. The Medipix 3.0 detector was controlled *via* the USB readout system developed at the Institute of Experimental and Applied Physics of the Czech Technical University (Czech Republic) (Vykydal *et al.*, 2006 ▶) under control of the *Pixelman* software (Turecek *et al.*, 2011 ▶). The measurements were taken in air under atmospheric pressure.

### 3.1. Resolution of beam position measurements   

**Figure 2:**
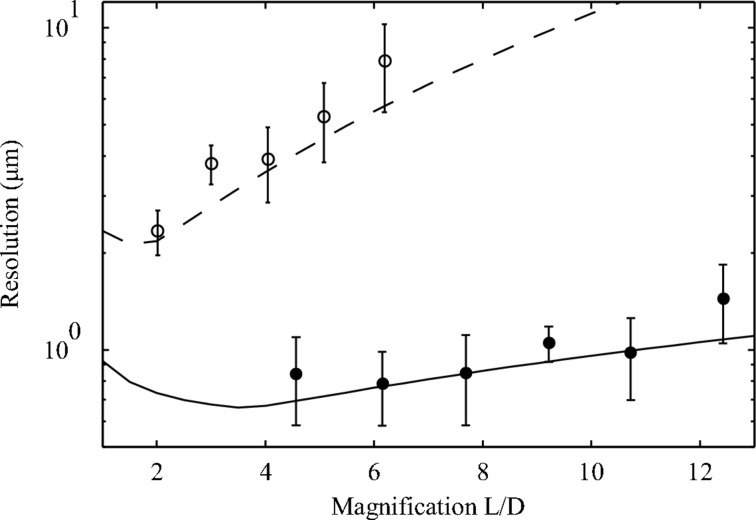
Resolution of horizontal beam displacement measurements *versus* XBI magnification as measured at beamline B16 using the XBI set-up with a constant exposure time of 1 s. The filled circles represent the measured data points taken using the XBI device set-up reported in this paper whereas the solid line represents calculated resolution values as predicted by our model (Kachatkou & van Silfhout, 2013 ▶). The open circles and the dashed line represent earlier measurements (Kachatkou & van Silfhout, 2013 ▶) and the corresponding calculated resolution values, respectively. See the text for details.

First, the performance of the XBI system in beam position measurements was evaluated. The XBI device was equipped with a 25 µm Kapton foil installed at 28.6° relative to the incident beam. A cross-shaped aperture with 100 µm slits was used. The exposure time was set to 1 s and kept constant during the measurements. The distance between the foil and the aperture was equal to 5 mm. The detector was moved into several positions at different distances from the aperture to obtain a range of magnification values. For each detector position, several image profiles were taken (Scott *et al.*, 2009 ▶). The measured image profiles were fitted with a Gaussian curve whose centre represented the position of the XBI image. The standard deviation values of image position measurements were then calculated and converted to the standard deviation of the beam displacement measurements as described by Kachatkou & van Silfhout (2013 ▶). We primarily focused on collecting data regarding the horizontal resolution of the device; the beam was found to be less stable in the vertical plane resulting in erroneous resolution values for the beam vertical position measurements. The results are shown in Fig. 2 ▶. The range of magnification values was limited by the constraints of the experimental set-up. Apart from the size of the cross-shaped aperture, both the XBI set-up described here and the experimental conditions were identical to ones reported by Kachatkou & van Silfhout (2013 ▶) where a custom-built indirect X-ray detector and a larger cross-shaped aperture with 200 µm slits were used. For comparison, Fig. 2 ▶ includes the resolution of beam displacement measurements obtained with that set-up (Kachatkou & van Silfhout, 2013 ▶).

### 3.2. Beam size measurements   

The performance of the XBI instrument in beam size measurements for micro-focused beams was studied by scanning the XBI set-up along the beam and taking several XBI images at each position. We aimed to improve the imaging characteristics of the lensless camera by opting for smaller apertures than in the measurements described above. First, a 25 µm-diameter circular aperture was used with the XBI magnification set to 5, which resulted in XBI images spreading over only 4–5 pixels in the image direction corresponding to the horizontal direction across the beam cross section. In the direction corresponding to the vertical direction across the beam cross section, the XBI images were significantly larger due to the foil effect (see Fig. 1 ▶). A 125 µm Kapton foil installed at 21° relative to the incident beam was used in these measurements to boost the intensity of the scattered X-rays. When using an exposure time of 8 s, the average intensity of the XBI image in the near focus region of the CRL was about 8 counts. For the second scan, a 50 µm circular aperture and XBI magnification of 6.5 were used to increase the spatial extent of the XBI image to about 9 pixels in the horizontal direction while maintaining image intensity at an acceptable level of 2 counts per pixel on average with the exposure time reduced to 2 s.

**Figure 3:**
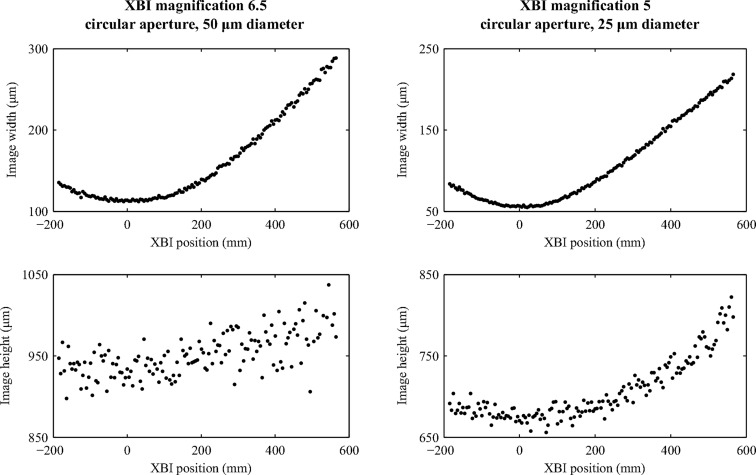
Gaussian width of XBI profiles acquired in different positions along the focused beam that corresponds to the beam size in the horizontal (top row) and vertical (bottom row) directions. The two sets of data are shown for two combinations of device magnification and aperture size as indicated by the captions of the corresponding columns. Other XBI parameters are given in the text.

Fig. 3 ▶ shows how the XBI image profile size (fitted Gaussian width) changes along the beam near the focal point of the CRL. The origin of the coordinate system is located at the point of the minimum of the calculated beam size.

**Figure 4:**
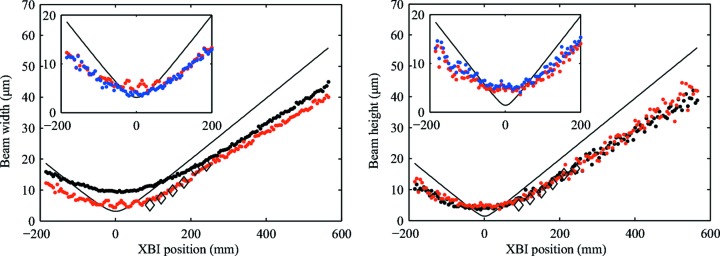
Beam size at different positions along the beam as measured near the focus by the XBI system with the direct detector (magnification of 5: black dots; magnification of 6.5: red dots) and the wire scanner (diamonds). The inset compares beam size in the near-focus region as measured for a magnification of 6.5 using images reconstructed on the normal (55 µm × 55 µm pixels, red dots) and oversampled (27.5 µm × 27.5 µm pixels, blue dots) grid (see discussion). The solid lines correspond to the theoretically predicted values.

To allow for a direct comparison with the expected beam size, we have plotted the calculated beam size and the geometry-corrected measurements of the beam size obtained using the method described in the previous section. The results are shown in Fig. 4 ▶.

A wire scanner consisting of two crossed 50 µm-thick gold wires installed in front of an X-ray-sensitive photodiode was used to provide a reference for our beam size measurements. The scanner was placed at six different locations along the beam path and in each location the vertical and horizontal beam profiles were obtained by scanning the wires across the beam (while keeping the photodiode stationary) and subsequent differentiating the collected measurements of beam intensity *versus* wire position. The width of the Gaussian fit of these profiles provided the measure of the beam size. The results of these measurements are included in Fig. 4 ▶.

Initially, oscillations of beam width measurements were observed near the point of best focus using a magnification of 6.5 (Fig. 4 ▶). In our experiments the beam was not parallel to the scan direction of the XBI device. Therefore, at each XBI location, the beam image was shifted by a fraction of a pixel relative to the image taken at the previous XBI location. For the values of beam sizes multiplied by the magnification factor that are comparable with the detector pixel size, the image becomes undersampled so that, when its centre is between two pixels, its size appears to be almost twice as large compared with when its centre is in the middle of the pixel. This effect can be reduced by performing image reconstruction on a grid oversampled using, for example, the nearest-neighbour method (Hanisch *et al.*, 2012 ▶). The beam width measurements obtained using beam cross-sectional images reconstructed on the grid with a step equal to and reduced by a factor of two relative to the Medipix 3.0 pixel size are compared in the insets of Fig. 4 ▶. The oversampling removes the oscillations of the measured beam size as shown in the beam width plot. However, it may also result in a slight overestimation of the beam size values in the near focus region due to a spatial expansion of the signal produced by the nearest-neighbour oversampling technique in the presence of the broad and slowly changing XBI PSF.

## 4. Discussion   

The test system used in this work was capable of providing position measurements of the micro-focused beam with a resolution of 0.78 µm r.m.s. (Fig. 2 ▶). The comparison with the previously reported results obtained in the same experiments but using the indirect detector based on an off-the-shelf high-performance CMOS sensor provides the experimental evidence to the theoretical argument presented by Kachatkou & van Silfhout (2013 ▶) that the XBI device resolution clearly benefits from ‘noiseless’ X-ray detection. An almost three-fold improvement in resolution was achieved with the single-photon-counting detector whereas the expected improvement from using a cross-shaped aperture with a more optimal slit width (100 µm instead of 200 µm) is only about 20% (Kachatkou & van Silfhout, 2013 ▶). Note that the resolution performance of the XBI device with both detectors is in good agreement with the predicted values calculated using the approach described by Kachatkou & van Silfhout (2013 ▶).

The results presented in Fig. 3 ▶ suggest that XBI images can be used directly to monitor changes in the beam size, albeit with some limitations. The resolution of such measurements depends on the device magnification, detector PSF, aperture size, scattering foil thickness and tilt angle, and the XBI image SNR. The detector PSF, the aperture size and the scattering foil geometry determine the uncertainty in XBI image profile size measurements due to the XBI set-up being a non-ideal imaging system. For the Medipix 3.0 detector, the detector PSF is approximately the rectangular function with a spatial extent equal to the pixel size. Its effect can be reduced using higher XBI magnification values provided the image SNR remains sufficiently high. In this work the SNR was affected by the fundamental X-ray photon shot noise and the background signal due to the X-ray scattering by the air in the space between the foil and the detector. The effect of noise on profile size measurements can be evaluated following the approach similar to that used to predict the resolution of beam position measurements by Kachatkou & van Silfhout (2013 ▶). Using a smaller aperture and a thinner foil with a steeper tilt angle will improve the resolution of XBI images by reducing the spatial extent of the XBI PSF. However, the number of scattered X-ray photons that reach the detector and, consequently, the image SNR and the resolution of profile size measurements will also be reduced. It is, therefore, important to emphasize that the choice of the XBI device parameters is a compromise between the various device characteristics as well as the practical considerations. For example, when comparing two sets of the XBI image width measurements reported in Fig. 3 ▶, the system with the 25 µm circular aperture and XBI magnification of 5 demonstrates marginally higher resolution in the near-focus region (the more pronounced extremum) due to the smaller aperture size but the system with the 50 µm circular aperture and XBI magnification of 6.5 provides the better resolution for larger beam sizes due to its higher magnification (the steeper slope of the plotted curve). On the other hand, the measurements of the XBI image size in the vertical direction in Fig. 3 ▶ show that in both cases system performance suffers from higher noise levels and low sensitivity to changes in the beam size because of the extra image blur caused by the foil tilt (see Fig. 1 ▶). Furthermore, when using the larger 50 µm circular aperture, the increased image blur and the lower SNR make it difficult to detect any significant change in the vertical beam size as compared with the set-up with the 25 µm circular aperture.

As demonstrated by the data reported in Fig. 4 ▶, the proposed method for obtaining absolute measurements of the beam size provides significantly better results. The resolution of the beam size measurements in this case is determined mostly by the relation between the detector pixel size and the device magnification. The effect of this relation is clearly visible in the beam width plot in Fig. 4 ▶. The minimal beam width measured with a magnification of 5 is 9.2 µm r.m.s. whereas the theoretically predicted width of the focused beam is 3.1 µm r.m.s.. Using an XBI magnification of 6.5 results in a minimal measured beam width of about 3.8 µm r.m.s. In the vertical direction, the foil tilt provides the additional image magnification. Hence, the minimal measured beam size values in that direction are similar for both magnifications and close to the true beam size of approximately 3.5 µm r.m.s. (see the beam vertical size plot in Fig. 4 ▶). The measurements obtained using an XBI magnification of 6.5 are in good agreement with the results obtained with the wire scanner.

**Figure 5:**
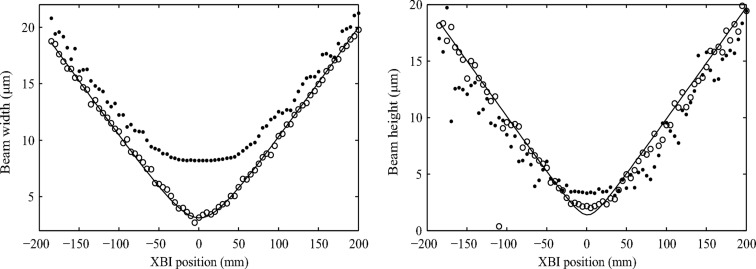
Beam size in different positions along the beam near the focus of the CRL as reconstructed from Monte Carlo simulations of XBI images for a magnification of 5 using a ‘noiseless’ detector with 55 µm × 55 µm pixels (black dots) and 7 µm × 7 µm pixels (circles); solid lines correspond to the theoretically predicted values. The other XBI parameters are the same as for the XBI instrument used to obtain results displayed in Fig. 4 ▶.

The theoretically predicted minimal beam size of 3.1 µm × 1.4 µm (h × v) is lower than measured. Also, the absolute value of the gradient of the theoretical beam size curves in Fig. 4 ▶ is higher. These discrepancies are explained by deviations of the actual beamline and CRL parameters from those used in our model and aberrations due to the CRL manufacturing tolerances. Although the effect of the XBI PSF is largely removed during image reconstruction, a very broad PSF, which is characteristic of the XBI devices, along with image noise cause reconstruction errors (Kachatkou *et al.*, 2013 ▶) and, consequently, could also result in slightly biased beam size values. Therefore, to verify the expected response of the XBI instrument, we conducted Monte Carlo simulations of our experiments. The incident X-ray beam was simulated for the same set of parameters as were used to calculate the theoretical curves in Fig. 4 ▶. The XBI images for each position of the XBI system along the beam were simulated for the parameters that described the XBI set-up with a magnification of 5, the same as for the corresponding measurements shown in Fig. 4 ▶. In order to investigate the effect of the pixel size on the resolution of beam size measurements, a set of XBI images using an imaginary hybrid pixel detector with a pixel size of 7 µm × 7 µm, which is typical for modern CCD and CMOS sensors (Sony ICX285AL; Coates *et al.*, 2009 ▶), was simulated. The beam images were reconstructed and beam size measurements were performed following the same procedure as for the experimental data. The results shown in Fig. 5 ▶ demonstrate that the image resolution is limited by the pixel size and prove that the results produced by our method of beam size measurements using the XBI instrument are correct.

The XBI device demonstrated a clear speed advantage over the wire scanner in our experiments. This is not surprising; beam size measurements using the wire scanner required two independent scans: one in the vertical and one in the horizontal direction. Owing to the relatively low intensity of the incident beam, each data point required 0.5 s of integration of the signal from the X-ray-sensitive diode. As a result, the total time spent on two scans of 160 data points each was over 2.6 min. In contrast, a single acquisition of beam position and beam size using the XBI device took slightly over 2 s. Even if several acquisitions per XBI position are necessary to improve the image SNR, the total measurement time with the XBI device was significantly shorter than with the wire scanner. The beam cross-sectional image reconstruction of direct detector 256 × 256 pixel images takes only 0.5 s for the Matlab script run on a standard office desktop computer equipped with an Intel Core 2 6600 dual core processor and 4 GB RAM.

Our experiments have demonstrated that despite the limitations caused by relatively large pixels the direct detector provides clear advantages. Higher resolution of beam position measurements and higher SNR of the XBI images are achievable with a hybrid pixel detector when compared with the indirect detector based on the off-the-shelf CMOS charge integrating sensor used in our previous work.

In this work we have shown that the recently introduced XBI device is an excellent tool for micro-focused beam characterization, and beam size measurements in particular. By carefully choosing the XBI magnification value, one can perform such measurements on micrometre-sized beams using X-ray detectors with relatively large pixels, such as the Medipix 3.0. The performance figures reported in this work do not represent the absolute maximum. As has been discussed above and also in our previous works, the performance of the XBI device depends on a number of parameters such as the intensity of the incident beam, acceptable detector exposure time, scatter foil characteristics and the aperture and magnification choice. These parameters must be carefully considered and decided upon with respect to the application requirements. As an added benefit, the geometry of the XBI set-up inherently provides one with the flexibility of being able to accommodate two lensless cameras that would image the scattering foil from above and below. These two cameras could use different detectors with different aperture shapes and sizes and XBI magnification settings so that two conflicting beam diagnostics tasks could be performed simultaneously and in an uncompromised way. For example, one can use a cross-shaped aperture with an optimal size to maximize the resolution and speed of beam displacement measurements (Kachatkou & van Silfhout, 2013 ▶), and a smaller aperture, higher magnification and a low-noise detector with slower readout times to obtain high-quality beam cross-section images and precise beam size measurements at a lower rate.
